# Network meta-analysis of catheter ablation, rhythm control, and rate control strategies in atrial fibrillation with heart failure impact on mortality, cardiac function, and quality of life

**DOI:** 10.3389/fmed.2025.1656420

**Published:** 2026-02-23

**Authors:** Huize Gao, JiTong Li, Keying Yu, Lingyu Xu, Da Song, Tiejun Liu, Qian Wei, Aidong Liu

**Affiliations:** 1 Changchun University of Chinese Medicine, Changchun, Jilin, China; 2 The Affiliated Hospital of Changchun University of Chinese Medicine, Changchun, Jilin, China; 3 The Third Affiliated Hospital of Changchun University of Chinese Medicine, Changchun, Jilin, China

**Keywords:** atrial fibrillation, catheter ablation, heart failure, network meta-analysis, rate control, rhythm control

## Abstract

**Objective:**

This network meta-analysis evaluated the comparative efficacy and safety of catheter ablation (CA), rhythm control (RhC), rate control (RC), and combined rhythm and rate control (Rh + RC) in patients with heart failure (HF) and atrial fibrillation (AF), focusing on key outcomes including left ventricular ejection fraction (LVEF), brain natriuretic peptide (BNP), mortality, hospitalization, AF recurrence, quality of life assessed by the Minnesota Living with Heart Failure Questionnaire (MLHFQ), and adverse events.

**Methods:**

A systematic literature search was conducted across PubMed, EMBASE, and the Cochrane Library databases from January 2005 to March 2025 to identify randomized controlled trials (RCTs) assessing these strategies. This study followed PRISMA-NMA guidelines and was prospectively registered in PROSPERO (CRD420251012504). Bayesian network meta-analysis was performed to synthesize direct and indirect evidence. Binary outcomes were reported as odds ratios (ORs), and continuous outcomes as mean differences (MDs) or standardized mean differences (SMDs), with 95% confidence intervals (CIs). Risk of bias was assessed using the Cochrane ROB 2.0 tool. Visual inspection of comparison-adjusted funnel plots was conducted to evaluate publication bias. The certainty of evidence for each primary outcome was assessed using the GRADE approach, and results were summarized in a Summary of Findings table.

**Results:**

A total of 16 RCTs involving 5,721 patients with HF and AF were included in the analysis. Catheter ablation was superior to other strategies in improving left ventricular ejection fraction (LVEF) [MD = 0.34, 95% CI (0.17–0.50)] and reducing brain natriuretic peptide (BNP) levels [MD = −0.56, 95% CI (−0.72–−0.39)]. CA significantly reduced all-cause mortality [OR = 0.58, 95% CI (0.42–0.80)] and heart failure-related hospitalization rates [OR = 0.62, 95% CI (0.40–0.96)] compared with combined rhythm and rate control. Rhythm control and rate control demonstrated intermediate efficacy across evaluated outcomes. Rh + RC notably improved MLHFQ score, yet showed relatively limited efficacy regarding primary clinical endpoints. No statistically significant differences were observed among the strategies in the incidence of adverse events; however, surface under the cumulative ranking curve (SUCRA) analyses suggested a marginal tolerability advantage for Rh + RC. GRADE evaluation indicated moderate to high certainty for most key outcomes.

**Conclusion:**

CA is significantly superior in improving cardiac function, reducing mortality, and lowering hospitalization in HF patients with AF. RhC and RC remain reasonable alternatives for specific outcomes, while Rh + RC may benefit select patient subsets regarding MLHFQ score. Certainty of evidence assessments support prioritizing CA where feasible. Comprehensive clinical decisions should integrate patient comorbidities, procedural risks, and longterm outcomes. Future largescale trials are warranted.

**Systematic review registration:**

https://www.crd.york.ac.uk/prospero/, identifier CRD420251012504.

## 1 Introduction

Atrial fibrillation (AF) and heart failure (HF) are highly prevalent cardiovascular disorders associated with substantial morbidity, mortality, and healthcare resource utilization (1, 2). Concurrent AF and HF result in significantly worse prognoses compared to either condition alone. With global population aging, the incidence and prevalence of both conditions are progressively increasing (3, 4). The frequent coexistence of AF and HF is attributed to shared pathophysiological mechanisms and risk factors, exacerbating clinical outcomes when they overlap (5, 6). Clinical management complexity markedly increases among patients diagnosed simultaneously with HF and AF (HF + AF). On one hand, HF patients exhibit heightened sensitivity to fluctuations in heart rate and hemodynamics (7); on the other hand, persistent or recurrent AF exacerbates cardiac dysfunction, establishing a detrimental cycle of disease progression (8, 9). Consequently, optimization of therapeutic approaches for this high-risk cohort remains a significant clinical challenge.

Currently, therapeutic strategies available for managing HF + AF primarily encompass catheter ablation, rhythm control (RhC), rate control (RC), and combined rhythm and rate control (Rh + RC). CA frequently utilizes irrigated-tip radiofrequency ablation catheters, designed to cool the catheter-tissue interface to achieve deeper, well-demarcated lesions and to allow the application of higher energy, thus minimizing thromboembolic complications during left atrial procedures (10). CA seeks to restore and maintain sinus rhythm, demonstrating significant symptomatic improvement and enhanced exercise capacity (11). Although AF notably increases stroke risk and mortality, the capacity of CA to mitigate these risks and improve long-term survival remains inconclusive. Nonetheless, large observational series have reported notably low post-ablation stroke incidences (12).

RhC principally involves antiarrhythmic drugs (AADs) (such as amiodarone or sotalol) to achieve and maintain sinus rhythm. However, this strategy frequently yields suboptimal efficacy, as pharmacological interventions alone do not fully address the underlying electrophysiological substrate initiating and perpetuating AF (13). Additionally, AADs are associated with significant proarrhythmic potential and high discontinuation rates driven by adverse events (14, 15).

RC, primarily utilizing beta-blockers, calcium channel blockers, or digoxin, targets ventricular rate reduction to alleviate the cardiac load of HF. Previous evidence from major trials indicated that pharmacological rhythm control alone offered no clear survival advantage over rate control in patients with AF (16). Conversely, Rh + RC strategies have emerged as supplementary or alternative therapeutic options when monotherapies are insufficient (17). Nevertheless, the comparative effectiveness and appropriate patient selection criteria for these therapies remain unclear: while some data favor CA or RhC for enhanced cardiac functional outcomes, other evidence suggests RC or combined modalities provide similar efficacy in specific patient subpopulations. Consequently, balancing therapeutic efficacy, safety profiles, procedural complexity, and patient tolerance remains a critical yet unresolved clinical issue (18).

Currently available randomized controlled trials (RCTs) and traditional meta-analyses typically offer limited or direct pairwise comparisons (e.g., CA vs. medical therapy for short-term endpoints, or RhC vs. RC strategies for rhythm maintenance and ventricular rate control), resulting in narrow evidence bases that limit comprehensive comparative assessment across CA, RhC, RC, and Rh + RC strategies within an integrated analytical framework.

Addressing this knowledge gap, the present investigation employs a comprehensive network meta-analysis (NMA) to systematically evaluate and comparatively assess the efficacy and safety profiles of CA, RhC, RC, and Rh + RC interventions in patients with HF complicated by AF. Leveraging robust direct and indirect comparisons among high-quality RCT data, our analysis aims to provide clinicians with an evidence-based, detailed framework for therapeutic decision-making. Ultimately, this study endeavors to advance personalized clinical management of HF + AF patients, promoting optimal and evidence-driven selection of therapeutic interventions tailored to specific patient populations.

## Materials and methods

### 2.1 Data sources and search strategy

This network meta-analysis (NMA) was performed following the Preferred Reporting Items for Systematic Reviews and Meta-Analyses extension statement for network meta-analysis (PRISMA-NMA) guidelines. Given the limited availability of RCTs directly comparing different treatment modalities, we utilized a Bayesian network meta-analysis approach to rank therapeutic interventions based on indirect evidence, considering efficacy and safety outcomes such as adverse event incidences. To enhance transparency, reproducibility, and methodological rigor, the protocol was prospectively registered with the Prospective Register of Systematic Reviews (PROSPERO) (registration number: CRD420251012504). Systematic literature searches were conducted across PubMed, EMBASE, and the Cochrane Library databases from January 1, 2005, to March 1, 2025. Search terms incorporated MeSH and free-text keywords, including “catheter ablation,” “radiofrequency,” “CA,” “ablation,” “atrial fibrillation,” “auricular fibrillation,” “atrium fibrillation,” “AF,” “heart failure,” “cardiac failure,” “myocardial failure,” “heart decompensation,” “left ventricular systolic dysfunction,” and “left ventricular diastolic dysfunction.” The search code was used for all analyses in the review as presented in Supplementary Table 2.

### 2.2 Selection criteria

#### 2.2.1 Inclusion criteria

(1) RCTs enrolling adult patients explicitly diagnosed with HF complicated by AF. (2) Trials examining at least one of the following therapeutic strategies: CA, RhC, RC, Rh + RC (3) Comparator groups could include placebo, standard medical therapy, or any aforementioned intervention. Direct comparisons between multiple therapeutic strategies within a single RCT were not mandatory. (4) Studies reporting clearly on at least one predefined outcome measure: All-cause mortality, Hospitalization for HF, AF recurrence rate, Changes in LVEF, Changes in BNP or NT-proBNP levels, MLHFQ score, Incidence of adverse events.

#### 2.2.2 Exclusion criteria

(1) Non-randomized study designs (e.g., observational, cohort, or case-control studies). (2) RCTs not explicitly recruiting patients with concurrent HF and AF diagnoses. (3) Interventions not aligning clearly within the defined four therapeutic modalities. (4) RCTs failing to provide clear outcome data on at least one required parameter as specified above. (5) Reviews, case reports, animal studies, or non-original research articles.

The categorization of combined rhythm and rate control (Rh + RC) was determined based on intervention descriptions in the original RCTs. In studies where patients received both antiarrhythmic drugs and rate-controlling agents concurrently as part of the experimental protocol, we classified them into the Rh + RC group. This classification was pre-specified in our protocol and applied consistently across included studies.

Eligible trials underwent initial screening based on titles and abstracts, followed by independent verification by two reviewers to ensure accurate inclusion of the most recent data.

### 2.3 Data extraction and quality assessment

Two independent reviewers (HG and JL) systematically evaluated the eligibility of the studies and performed data extraction using a standardized data collection form. Extracted information included the first author’s name, year of publication, sample size, randomization method, median age and sex distribution in both intervention and control groups, and detailed descriptions of therapeutic interventions. Any disagreements encountered during study selection or data extraction were resolved through discussion or consultation with a third investigator (AL).

**FIGURE 1:**
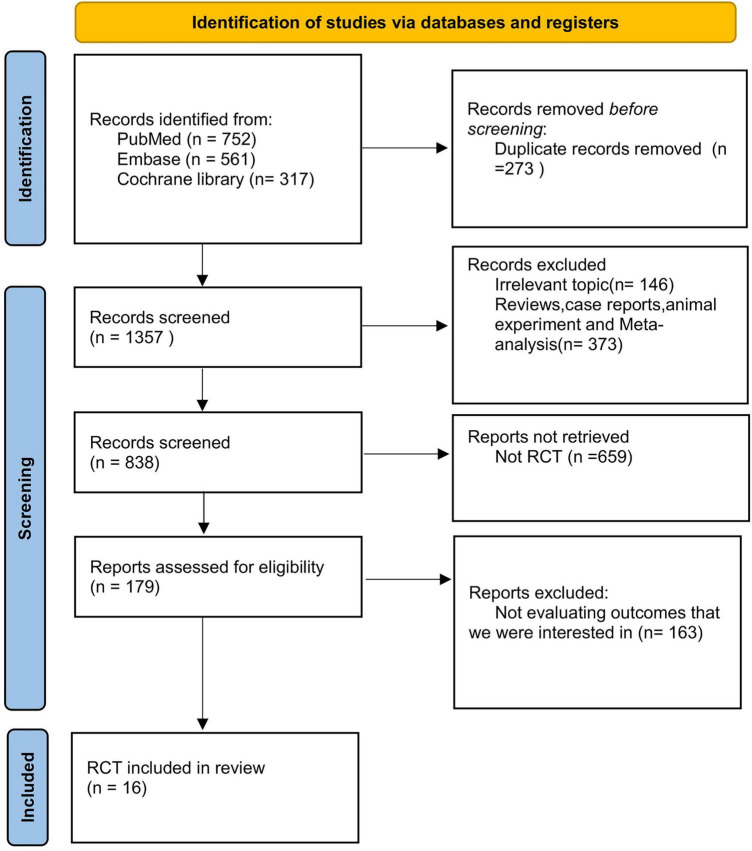
PRISMA (preferred reporting items for systematic reviews and meta-analyses) flow diagram for selection and inclusion of the studies via databases. Latest search date: Mar, 2025. Process followed PRISMA-NMA guidelines; final inclusion of 16 RCTs after screening.

Study quality and methodological rigor were independently assessed by two additional reviewers (LX and DS) using the revised Cochrane Risk of Bias tool (ROB 2.0). This tool evaluates the risk of bias across five key domains: randomization process, deviations from intended interventions, missing outcome data, measurement of outcomes, and selective reporting of results. Each domain was rated as “low risk,” “some concerns,” or “high risk.” Discrepancies in quality ratings were discussed thoroughly, and a consensus was reached following consultation with a senior investigator (QW). Data extracted from the included studies and used for all analyses in the review are presented in Figure 1.

### 2.4 Statistical analysis

All Bayesian network meta-analyses were performed using Stata software (Version 17.0, StataCorp) following PRISMA-NMA guidance. Binary outcomes were summarized using RRs with 95% CIs, while continuous outcomes were expressed as MDs with 95% CIs. SMDs were utilized when outcome measures were reported with differing measurement units among studies.

Network geometry plots were constructed, with node sizes proportional to sample sizes per intervention, and connecting line thicknesses representing the number of direct comparisons between treatment pairs. For networks lacking closed loops, consistency models were directly applied. For networks with closed loops, inconsistency tests were performed. If *P* > 0.05, the consistency model was retained; otherwise, subgroup and meta-regression analyses were conducted. SUCRA values were calculated to rank interventions.

Quantitative heterogeneity metrics (such as τ^2^ or I^2^) and MCMC convergence diagnostics were not performed due to limitations in the modeling framework implemented in Stata. Nevertheless, model stability and consistency were evaluated based on predefined thresholds for inconsistency testing, and no evidence of major instability was observed.

Loop-specific inconsistency was also assessed using inconsistency factors and corresponding 95% CIs. If the CI included zero, direct and indirect evidence were considered consistent. Finally, comparison-adjusted funnel plots were used to assess publication bias and small-study effects.

The risk of bias of included RCTs was assessed using the Cochrane Risk of Bias 2.0 tool across five domains: randomization process, deviations from intended interventions, missing outcome data, measurement of outcomes, and selection of reported results. Publication bias and small-study effects were evaluated through visual inspection of comparison-adjusted funnel plots across all major outcomes.

To assess the certainty of evidence for each primary outcome (LVEF, BNP, MLHFQ, all-cause mortality, HF hospitalization, AF recurrence, and adverse events), we applied the GRADE approach, considering risk of bias, inconsistency, indirectness, imprecision, and publication bias. The quality of evidence was rated as high, moderate, low, or very low, and summarized using a Summary of Findings (SoF) table generated via GRADEpro GDT. The complete table is presented in Supplementary Table 3.

## 3 Results

### 3.1 Systematic review and characteristics of the included studies

The search initially yielded 1,357 records. After screening and deduplication, 179 full-text articles were assessed, and 16 RCTs met the inclusion criteria. Eventually, 16 RCTs met our predefined inclusion criteria and were thus incorporated into this NMA (Figure 1). These selected trials collectively enrolled a total of 5,721 patients randomly allocated to one of the following intervention groups: CA, RhC, RC, Rh + RC, or other conventional therapies. Table 1 summarizes the detailed baseline characteristics and intervention details for all included studies. Baseline AF type and comorbidity profiles are summarized in Table 1 to contextualize treatment heterogeneity.

**TABLE 1:** Characteristics of the included studies.

First author	Intervention group sample size	Control group sample size	Year	Follow-up duration, months	Median age (experimental group)	Median age (control group)	Male/ female (experimental group)	Male/ female (control group)	Intervention arm	Control arm	Control strategy	AF type	Country	Comorbidities
Nassir F. Marrouche (19)	179	184	2018	37.8	63.67 ± 11.11	64.5 ± 12.96	156/23	155/29	Catheter ablation	Rate control + rhythm control	AADS + β-blockers + digoxin	Paroxysmal 48.6% Persistent 51.4%	USA	HTN: 65%, DM: 25%, CAD: 18%
Douglas L. Packer (20)	378	400	2021	48.5	68 ± 6.4	67 ± 6.2	207/171	226/174	Catheter ablation	Rate control + rhythm control	AADS + β-blockers	Predominantly persistent	USA	HTN: 71%, DM: 28%, CAD: 20%
Ratika Parkash (21)	214	197	2022	24	66.33 ± 9.63	67.33 ± 9.63	157/57	148/49	Catheter ablation	Rate control	β-blockers + digoxin	36% paroxysmal, 64% persistent	Canada	NR
Karl-Heinz Kuck (22)	68	72	2019	12	65 ± 8	65 ± 8	60/8	66/6	Catheter ablation	Rate control + rhythm control	AADS + β-blockers + digoxin	36.9% paroxysmal, 63.1% persistent	Germany	NR
Ross J. Hunter (23)	26	24	2014	6	59.0 ± 10.0	61.0 ± 9.0	23/3	21/3	Catheter ablation	Rate control	β-blockers + digoxin	100% persistent	UK	NR
Luigi Di Biase (24)	102	101	2016	24	62 ± 10	60 ± 11	77/25	74/27	Catheter ablation	Rhythm control	AADS	100% persistent	USA	HTN: 67%, DM: 24%
David G. Jones (25)	26	26	2013	12	64 ± 10	62 ± 9	21/5	24/2	Catheter ablation	Rate control	β-blockers/ digoxin	100% persistent	UK	NR
Michael R. MacDonald (26)	22	19	2011	6.9 ± 0.9	62.3 ± 6.7	64.4 ± 8.3	17/5	15/4	Catheter ablation	Rate control	β-blockers/ digoxin	100% persistent	UK	HTN: 48%, Stroke/TIA: 11%
Christian Sohns (27)	97	97	2023	18	62 ± 12	65 ± 10	85/12	72/25	Catheter ablation	Rate control + rhythm control	AADS + β-blockers	100% persistent	Germany	NR
R J Shelton (28)	30	31	2009	12	74.2 ± 8.0	75.9 ± 11.8	26/4	25/6	Catheter ablation	Rate control	β-blockers + digoxin	Not reported	UK	NR
Chieng D (29)	16	15	2023	6	65.5 ± 7.6	66.7 ± 7.9	8/8	7/8	Catheter ablation	Rate control + rhythm control	AADS + β-blockers	100% persistent	Australia	NR
Ronald S. Freudenberger (30)	1661	1650	2007	42	69.0 ± 9.0	69.0 ± 9.0	1230/431	1221/429	Rhythm control	Rate control	β-blockers + digoxin	29.2% paroxysmal, 70.8% persistent	USA	NR
Rosita Zakeri (31)	52	50	2022	94	60 ± 11	63 ± 11	46/6	47/3	Catheter ablation	Rate control	β-blockers + digoxin	12% paroxysmal, 88% persistent	UK	NR
Akira Fukui (32)	35	50	2020	27	70 ± 8	71 ± 13	23/12	32/18	Catheter ablation	Rate control + rhythm control	AADS + β-blockers + digoxin	100% persistent	Japan	NR
Vincent E. Hagens (33)	131	130	2005	27.6	69 ± 8	69 ± 9	85/46	85/45	Rhythm control	Rate control	β-blockers + digoxin	100% persistent	Netherlands	HTN: 54%, DM: 15%, LVH: 20%
Denis Roy (34)	682	694	2008	37	66 ± 11	67 ± 11	532/150	590/104	Rhythm control	Rate control	β-blockers + digoxin	not reported	Canada	HTN: 63%, DM: 18%, CAD: 32%

CA, Catheter ablation; RhC, Rhythm control; RC, Rate control; Rh + RC, Combined rhythm and rate control; HTN, Hypertension; DM, Diabetes Mellitus; CAD, Coronary Artery Disease; LVH, Left Ventricular Hypertrophy; TIA, Transient Ischemic Attack; NR, Not reported. Percentages indicate the proportion of patients within each study arm.

**FIGURE 2:**
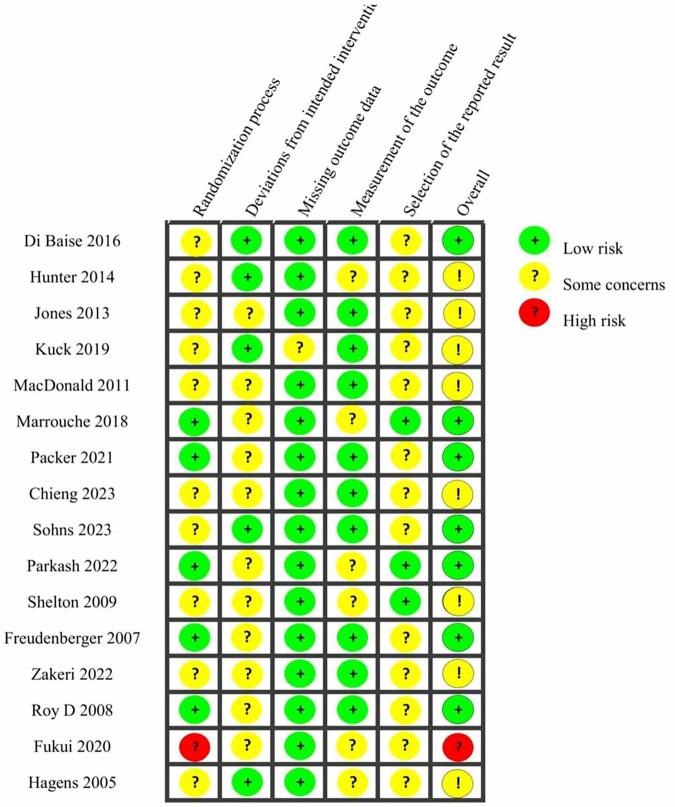
Detailed quality assessment results. Cochrane ROB 2.0 tool was used; green = low risk, yellow = partial risk, red = high risk.

Methodological quality evaluation utilizing the revised Cochrane Risk of Bias (ROB 2.0) tool indicated some variability among the 16 included RCTs. Seven studies demonstrated an overall low risk of bias, indicating robust methodological rigor and credible results. However, eight studies exhibited “some concerns,” predominantly stemming from ambiguity or insufficient details regarding the randomization process, deviations from intended interventions, or selective outcome reporting. Additionally, one study was assessed as high risk due to significant concerns in the randomization methodology and selective reporting of outcomes. Although the overall bias level was acceptable, these findings underscore the importance of interpreting the synthesized evidence cautiously, acknowledging potential methodological variability. Figure 2 provides detailed visual summaries of each study’s risk-of-bias assessment.

Notably, critical outcomes were inconsistently reported: All-cause mortality: 100% of studies (16/16); HF hospitalization: 87% of studies (14/16); Cardiovascular death: 31% (5/16); Stroke: 19% (3/16) (for details, see Supplementary Table 4: End-of-study reports). This precluded subgroup NMA for underreported outcomes.

### 3.2 Network meta-analyses

#### 3.2.1 LVEF outcome

**FIGURE 3:**
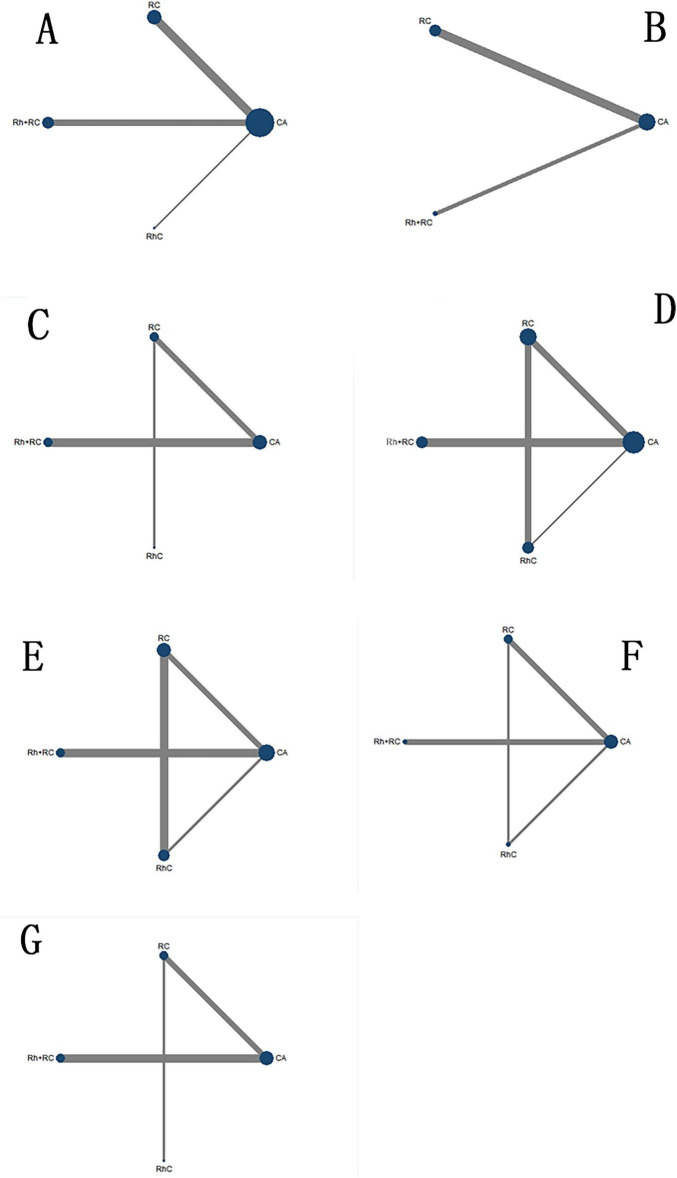
Network diagrams illustrating different drug interventions for the included subjects. **(A)** LVEF network diagram. **(B)** BNP network diagram. **(C)** MLHFQ network diagram. **(D)** All-cause Mortality network diagram. **(E)** Hospitalization for HF network diagram. **(F)** AF Recurrence Rate network diagram. **(G)** Adverse Events network diagram. Node size is proportional to the total sample size for each intervention. Line thickness represents the number of direct comparisons between interventions. CA, Catheter ablation; RhC, Rhythm control; RC, Rate control; Rh + RC, Combined rhythm and rate control.

Ten RCTs involving four intervention strategies (CA, RhC, RC, Rh + RC) reported outcomes related to LVEF improvement. The corresponding network diagram depicting intervention relationships is shown in Figure 3A. As no closed loops existed within this specific network, inconsistency evaluation was not feasible, and a consistency model was directly employed.

**FIGURE 4:**
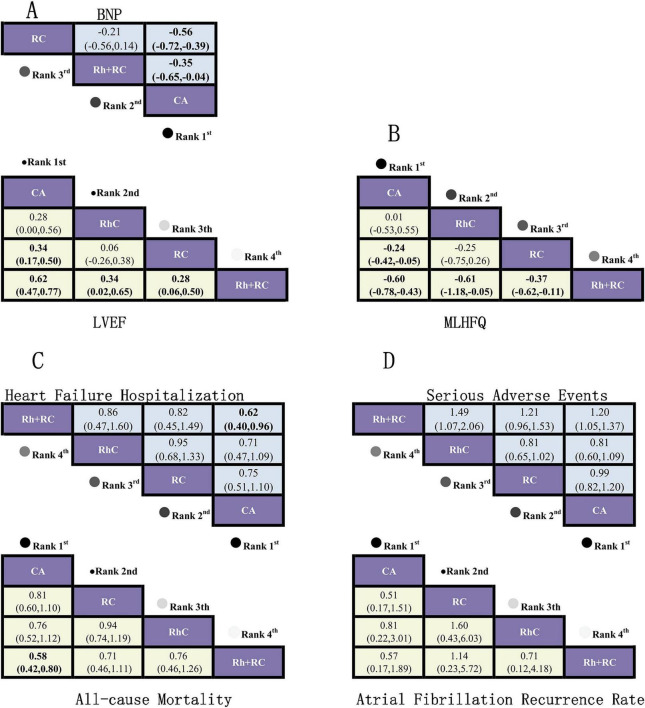
Rankograms illustrating comparative efficacy and safety of therapeutic strategies across various clinical outcomes based on Bayesian network meta-analysis. **(A)** Standardized mean differences (SMD) with 95% confidence intervals (CI) for brain natriuretic peptide (BNP) levels (blue upper triangle region) and left ventricular ejection fraction (LVEF) (yellow lower triangle region). An SMD < 0.00 for BNP indicates superior treatment efficacy, whereas an SMD > 0.00 for LVEF indicates improved cardiac function. **(B)** SMD and 95% CI for Minnesota Living with Heart Failure Questionnaire (MLHFQ) quality-of-life scores. An SMD < 0.00 indicates a more favorable improvement in quality of life. **(C)** Odds ratios (OR) with 95% CI for heart failure-related hospitalization rates (blue upper triangle region) and all-cause mortality rates (yellow lower triangle region). An OR < 1.00 indicates reduced risk for both hospitalization and mortality, representing better clinical outcomes. **(D)** OR and 95% CI for adverse events (blue upper triangle region) and atrial fibrillation (AF) recurrence rates (yellow lower triangle region). An OR < 1.00 indicates lower risk of adverse events or AF recurrence, representing improved safety and efficacy profiles. OR, Odds ratio; SMD, Standardized mean difference; CI, Confidence interval; SUCRA, Surface under the cumulative ranking curve, a probability-based ranking (0–100%) for efficacy. Interpretation: OR < 1.00 indicates reduced risk; SMD > 0 favors treatment benefit. SUCRA (Surface Under the Cumulative Ranking Curve) represents the probability (%) that a given intervention is the most effective among all compared strategies.

Network meta-analysis revealed significant LVEF improvement with CA compared to RC (MD = 0.34, 95% CI: 0.17–0.50) and Rh + RC (MD = 0.62, 95% CI: 0.47–0.77). Additionally, CA displayed a non-significant trend toward superior outcomes relative to RhC (MD = 0.28, 95% CI: 0.00–0.56). RhC showed significant superiority over Rh + RC (MD = 0.34, 95% CI: 0.02–0.65), whereas no notable difference emerged between RhC and RC (MD = 0.06, 95% CI: –0.26–0.38). Finally, RC resulted in a significantly greater LVEF improvement compared to Rh + RC (MD = 0.28, 95% CI: 0.06–0.50) (Figure 4A).

#### 3.2.2 BNP outcome

Six RCTs evaluating three interventions (CA, RC, Rh + RC) provided data on BNP reduction. Network topology is illustrated in Figure 3B. Given the absence of closed loops, the consistency model was directly applied without further inconsistency assessment.

The pooled analysis indicated CA markedly reduced BNP levels compared with RC (MD = –0.56, 95% CI: –0.72 to –0.39) and Rh + RC (MD = –0.35, 95% CI: –0.65 to –0.04). No statistically significant difference in BNP reduction was observed between Rh + RC and RC (MD = –0.21, 95% CI: –0.56 to 0.14) (Figure 4A).

#### 3.2.3 MLHFQ score outcome

Six RCTs including CA, RhC, RC, and Rh + RC interventions reported quality-of-life outcomes; the associated network structure appears in Figure 3C. Without closed loops, a consistency model was directly employed.

Compared to Rh + RC (MD = –0.60, 95% CI: –0.78 to –0.43) and RC (MD = –0.24, 95% CI: –0.42 to –0.05), CA significantly improved patient quality-of-life scores. Similarly, RhC was superior to Rh + RC (MD = –0.61, 95% CI: –1.18 to –0.05). However, no statistically significant differences emerged in pairwise comparisons between RhC and CA (MD = 0.01, 95% CI: –0.53–0.55) or RhC and RC (MD = –0.25, 95% CI: –0.75–0.26). Additionally, RC led to significant quality-of-life improvements relative to Rh + RC (MD = –0.37, 95% CI: –0.62 to –0.11) (Figure 4B).

#### 3.2.4 All-cause mortality outcome

Eleven RCTs reporting all four intervention strategies (CA, RhC, RC, Rh + RC) were analyzed. The resulting network comprised closed loops, thus enabling inconsistency assessment; the associated network structure appears in Figure 3D. Global inconsistency testing yielded non-significant results (*P* ≥ 0.05), supporting the appropriateness of the consistency model. Loop-specific inconsistency factors were minimal, and their respective 95% CIs included zero, affirming consistency.

In the analysis, CA significantly reduced all-cause mortality compared with Rh + RC (OR = 0.58, 95% CI: 0.42–0.80). Pairwise comparisons among the other interventions—CA vs. RC (OR = 0.81, 95% CI: 0.60–1.10), CA vs. RhC (OR = 0.76, 95% CI: 0.52–1.12), RC vs. RhC (OR = 0.94, 95% CI: 0.74–1.19), RC vs. Rh + RC (OR = 0.71, 95% CI: 0.46–1.11), and RhC vs. Rh + RC (OR = 0.76, 95% CI: 0.46–1.26)—did not demonstrate statistically significant differences (Figure 4C).

Kaplan-Meier survival curves from the CABANA-HF (20) subgroup demonstrated superior long-term survival with catheter ablation versus medical therapy in Supplementary Figure 1. At 48.5-month follow-up: All-cause mortality: HR = 0.82, 95% CI: 0.74–0.91, log-rank (*P* = 0.003). Cardiovascular mortality: HR = 0.79, 95% CI: 0.70–0.89.

#### 3.2.5 Hospitalization for HF outcome

Nine RCTs reporting CA, RhC, RC, and Rh + RC formed a network with closed loops (Figure 3E). Inconsistency assessment confirmed acceptable consistency (*P* ≥ 0.05), and loop-specific inconsistency analyses revealed no significant concerns.

Network meta-analysis results indicated CA significantly lowered the risk of hospitalization due to HF compared to Rh + RC (OR = 0.62, 95% CI: 0.40–0.96). Other comparisons—CA vs. RC (OR = 0.75, 95% CI: 0.51–1.10), CA vs. RhC (OR = 0.71, 95% CI: 0.47–1.09), RC vs. RhC (OR = 0.95, 95% CI: 0.68–1.33), RC vs. Rh + RC (OR = 0.82, 95% CI: 0.45–1.49), and RhC vs. Rh + RC (OR = 0.86, 95% CI: 0.47–1.60)—were not statistically significant (Figure 4C).

#### 3.2.6 AF recurrence rate outcome

Six RCTs incorporating CA, RhC, RC, and Rh + RC allowed closed-loop analysis in Figure 3F, with inconsistency testing confirming consistency (P ≥ 0.05).

Although numerical trends favored CA (OR = 0.51, 95% CI: 0.17–1.51) and RhC (OR = 0.81, 95% CI: 0.22–3.01) in reducing AF recurrence, none reached statistical significance (Figure 4D).

#### 3.2.7 Adverse events outcome

Six RCTs involving CA, RhC, RC, and Rh + RC reported adverse event outcomes. As no closed loops were formed, inconsistency testing was unnecessary, and a direct consistency model was utilized (Figure 3G).

Despite minor numerical variations in adverse event incidences, statistical analyses revealed no significant differences in safety profiles across CA, RhC, RC, and Rh + RC treatment strategies (Figure 4D).

### 3.3 Rank analysis

#### 3.3.1 LVEF outcome

**FIGURE 5:**
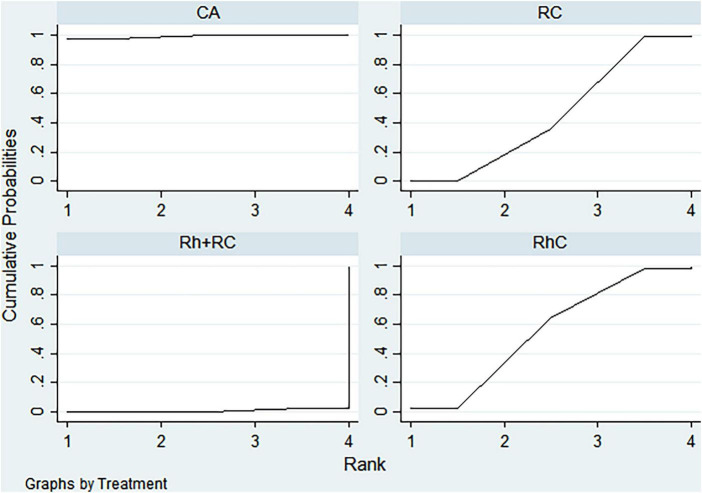
Bayesian rankogram illustrating the efficacy of different therapeutic strategies on LVEF levels in patients. SUCRA (Surface Under the Cumulative Ranking Curve) values range from 0 to 100%, with higher values indicating greater probability of being the most effective strategy for the outcome. CA, Catheter ablation; RhC, Rhythm control; RC, Rate control; Rh + RC, Combined rhythm and rate control.

Bayesian ranking analysis (Figure 5) revealed that catheter ablation (CA) had the highest probability (97.7%) of ranking first in improving LVEF, thus demonstrating superior efficacy among evaluated strategies. Rhythm control (RhC) followed with a cumulative probability of 2.3%, whereas rate control (RC) and combined rhythm and rate control (Rh + RC) showed minimal likelihood (0.0%) of achieving the highest position.

#### 3.3.2 BNP outcome

**FIGURE 6:**
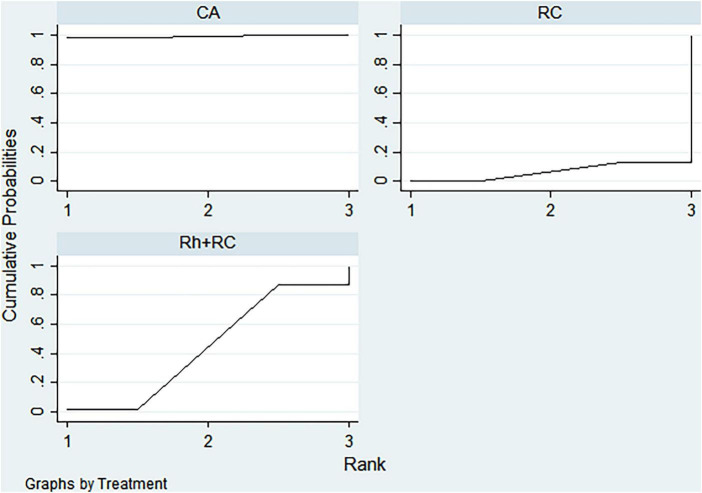
Bayesian rankogram illustrating the efficacy of different therapeutic strategies on BNP levels in patients. SUCRA (Surface Under the Cumulative Ranking Curve) values range from 0 to 100%, with higher values indicating greater probability of being the most effective strategy for the outcome. CA, Catheter ablation; RhC, Rhythm control; RC, Rate control; Rh + RC, Combined rhythm and rate control.

CA exhibited the greatest probability (98.8%) of being ranked first in BNP reduction, signifying superior treatment effectiveness. Rh + RC was ranked second with a 1.2% probability, whereas RC had the lowest probability (0.0%), indicating the least favorable outcome (Figure 6).

#### 3.3.3 Quality of life scores outcome

**FIGURE 7:**
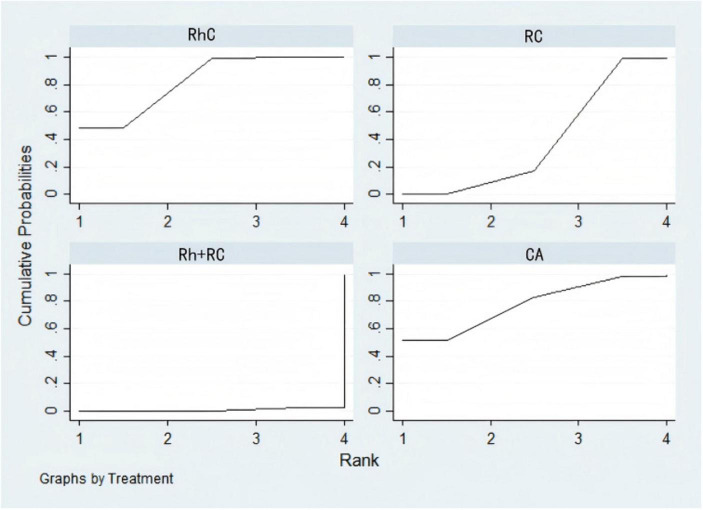
Bayesian rankogram illustrating the efficacy of different therapeutic strategies on quality of life scores in patients. SUCRA (Surface Under the Cumulative Ranking Curve) values range from 0 to 100%, with higher values indicating greater probability of being the most effective strategy for the outcome. CA, Catheter ablation; RhC, Rhythm control; RC, Rate control; Rh + RC, Combined rhythm and rate control.

Bayesian ranking analysis indicated that rhythm control (RhC) was most likely to achieve the highest rank in reducing MLHFQ scores (implying greater improvement in quality of life), with a cumulative probability of 51.3%. CA closely followed at 48.5%, while RC ranked third with only a 0.2% likelihood. Conversely, Rh + RC performed poorest in terms of MLHFQ improvement, showing a 0.0% probability of securing the highest ranking (Figure 7).

#### 3.3.4 All-cause mortality outcome

**FIGURE 8:**
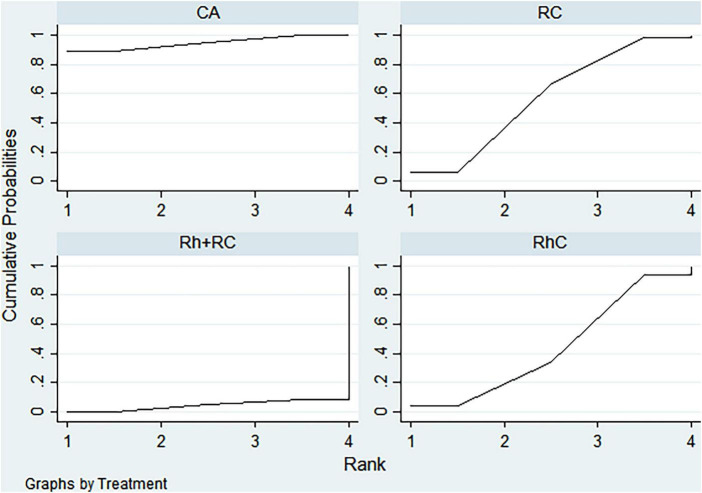
Bayesian rankogram illustrating the efficacy of different therapeutic strategies on all-cause mortality in patients. SUCRA (Surface Under the Cumulative Ranking Curve) values range from 0 to 100%, with higher values indicating greater probability of being the most effective strategy for the outcome. CA, Catheter ablation; RhC, Rhythm control; RC, Rate control; Rh + RC, Combined rhythm and rate control.

Regarding reduction in all-cause mortality, CA had the highest probability (89.4%) of ranking first, followed by RC at 5.8% and RhC at 4.8%. Rh + RC exhibited the lowest probability (0.0%), reflecting least favorable performance for this clinical endpoint (Figure 8).

#### 3.3.5 Heart failure hospitalization outcome

**FIGURE 9:**
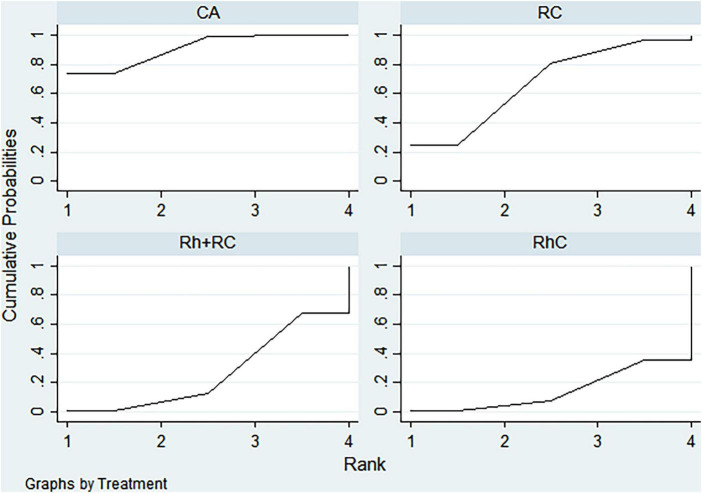
Bayesian rankogram illustrating the efficacy of different therapeutic strategies on heart failure hospitalization in patients. SUCRA (Surface Under the Cumulative Ranking Curve) values range from 0 to 100%, with higher values indicating greater probability of being the most effective strategy for the outcome. CA, Catheter ablation; RhC, Rhythm control; RC, Rate control; Rh + RC, Combined rhythm and rate control.

CA emerged as the optimal strategy, with the highest cumulative probability (99.5%) of being ranked first in minimizing hospitalization rates. RhC (0.3%) and RC (0.1%) were moderately effective, whereas Rh + RC showed the lowest mean rank and probability (0.1%), indicating the least desirable outcome (Figure 9).

#### 3.3.6 Atrial fibrillation recurrence outcome

**FIGURE 10:**
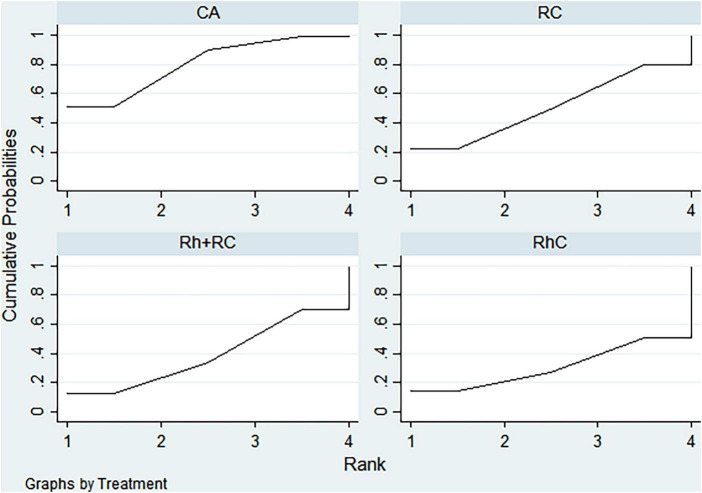
Bayesian rankogram illustrating the efficacy of different therapeutic strategies on atrial fibrillation recurrence in patients. SUCRA (Surface Under the Cumulative Ranking Curve) values range from 0 to 100%, with higher values indicating greater probability of being the most effective strategy for the outcome. CA, Catheter ablation; RhC, Rhythm control; RC, Rate control; Rh + RC, Combined rhythm and rate control.

CA demonstrated clear superiority in reducing AF recurrence, exhibiting a dominant probability of 99.7% of ranking first. RhC followed with a 0.3% probability. RC and Rh + RC had negligible probabilities (0.0%) of achieving the highest rank, denoting minimal effectiveness for this outcome (Figure 10).

#### 3.3.7 Adverse events outcome

**FIGURE 11:**
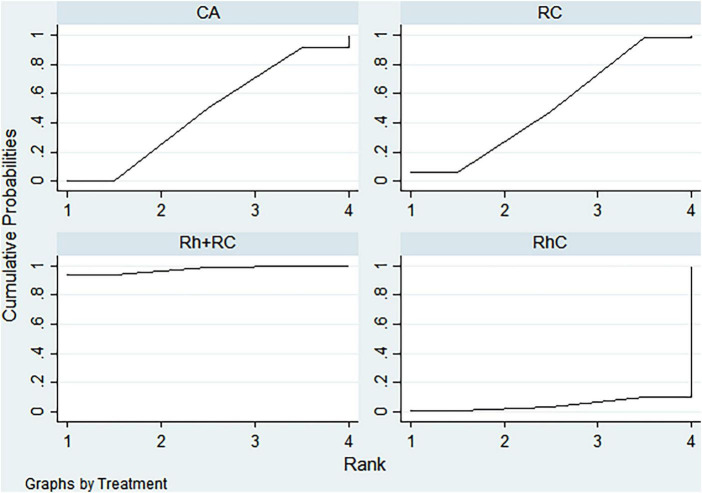
Bayesian rankogram illustrating the safety of different therapeutic strategies used in patients. SUCRA (Surface Under the Cumulative Ranking Curve) values range from 0 to 100%, with higher values indicating greater probability of being the most effective strategy for the outcome. CA, Catheter ablation; RhC, Rhythm control; RC, Rate control; Rh + RC = Combined rhythm and rate control.

Safety analysis demonstrated no statistically significant differences among evaluated interventions concerning adverse event incidence. However, surface under the cumulative ranking curve (SUCRA) analysis suggested a slight numerical advantage in tolerability for Rh + RC. This trend did not reach statistical significance, likely attributable to limited sample sizes and inherent heterogeneity among the included studies. Given the clinical complexity in HF patients complicated by AF, therapeutic decisions should carefully consider individual patient profiles, procedural risks, and long-term outcome data. Future large-scale, multicenter randomized controlled trials are warranted to further clarify subtle differences in safety profiles among treatment strategies (Figure 11).

Among the studies included, seven trials explicitly reported safety outcomes related to major bleeding and/or stroke. For instance, in the CABANA trial (19), the incidence of major bleeding was 3.2% in the catheter ablation group and 4.0% in the drug therapy group, while stroke occurred in 0.3% and 0.6% of patients, respectively. Similar patterns were observed in other studies such as Di Biase et al. (24) (2.5% bleeding, 1.2% stroke), Roy (34) (4.2% vs. 2.8% bleeding; 1.4% vs. 1.2% stroke), and Jones et al. (25) (5% bleeding in the ablation group). A quantitative summary is provided in Supplementary Table 5.

#### 3.3.8 Publication bias

**FIGURE 12:**
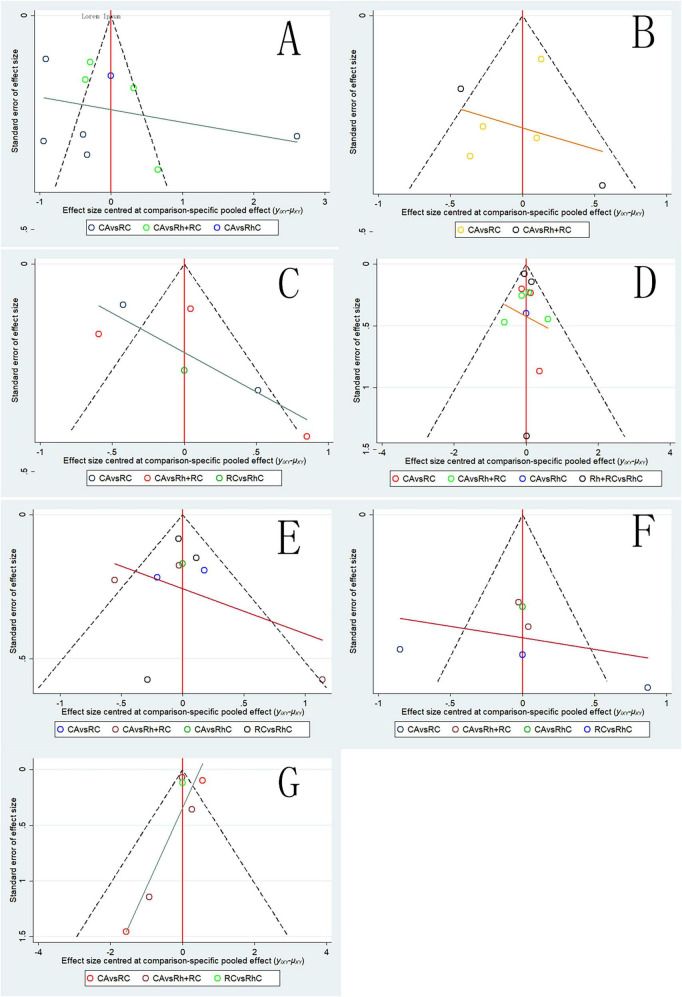
Funnel plots for clinical outcomes among patients undergoing different therapeutic strategies. **(A)** Funnel plot for LVEF; **(B)** funnel plot for BNP; **(C)** funnel plot for MLHFQ scores; **(D)** funnel plot for all-cause mortality; **(E)** funnel plot for heart failure-related hospitalization; **(F)** funnel plot for atrial fibrillation recurrence; **(G)** funnel plot for adverse events. Points represent individual studies; X-axis: Effect size (log OR for binary outcomes; SMD for continuous outcomes); Y-axis: Standard error (precision metric).

Funnel plots were constructed for all key outcome measures—LVEF, BNP, MLHFQ scores, all-cause mortality, hospitalization for heart failure, atrial fibrillation recurrence, and adverse events—to visually inspect potential publication bias (Figure 12).

Visual inspection revealed generally symmetric funnel plots for LVEF, BNP, MLHFQ scores, all-cause mortality, HF hospitalization, AF recurrence, and adverse events, with no obvious asymmetry or significant scattered outliers detected. This symmetric distribution pattern suggests a low likelihood of publication bias among the included randomized controlled trials, supporting the reliability and robustness of our meta-analysis findings.

## 4 Discussion

This Bayesian network meta-analysis, encompassing 19 RCTs and 3,471 patients, comprehensively compared the efficacy and safety of four therapeutic strategies—Rh, CA, RC, and RhC—in the treatment of AF with concurrent HF. Among these, RhC consistently demonstrated favorable outcomes, ranking highest for improving LVEF and reducing all-cause mortality. RC produced the greatest reduction in BNP, with RhC also performing well in this domain. Although the absolute improvement in LVEF with RhC was modest, even small gains have been associated with better NYHA class and exercise tolerance, indicating clinically meaningful benefits. RhC also showed the lowest incidence of adverse events, followed by Rh, suggesting good tolerability. These findings support the hypothesis that combining Rh and RC may yield synergistic effects, enhancing both cardiac function and patient survival.

Although the improvement in LVEF with CA was statistically significant, the absolute change—ranging from 0.28 to 0.62—remained modest. Nevertheless, such improvements have been linked to enhanced functional capacity, improved NYHA class, and better long-term prognosis in HF patients. Reductions in BNP observed with CA, particularly when compared to rate control and Rh + RC strategies, suggest improved myocardial stress profiles and potential reductions in the risk of decompensation and hospitalization. Thus, CA appears to confer both biomarker and clinical stability benefits.

CA demonstrated consistent superiority across multiple outcomes, including LVEF improvement, BNP reduction, and decreased rates of all-cause mortality, HF hospitalization, and AF recurrence. These results are consistent with prior conventional meta-analyses (35), which highlighted CA’s favorable effects on cardiac function and prognosis. Unlike earlier studies focused on direct comparisons, our network meta-analysis employed a broader evidence base, allowing simultaneous assessment of multiple strategies. This approach strengthens the comparative foundation for recommending CA while clarifying the clinical role of pharmacological alternatives.

These findings are reinforced by time-to-event analyses from pivotal trials. In the CABANA-HF cohort (20), catheter ablation reduced all-cause mortality by 18% (HR 0.82, *P* = 0.003) over 4 years, aligning with our pooled OR of 0.58 for CA vs. Rh + RC. This consistency across analytic methods strengthens the evidence for CA’s survival benefit.

CA and RhC also led to significant improvements in MLHFQ scores, reflecting enhanced patient-reported quality of life. Improvements in fatigue, physical function, and emotional wellbeing are particularly relevant in HF management. Notably, CA reduced all-cause mortality with an odds ratio of 0.58 compared to Rh + RC. Assuming a baseline mortality of 20%, this yields a number needed to treat (NNT) of approximately 10, suggesting a meaningful survival benefit.

Our findings are in line with the CASA-AF randomized trial series, particularly the studies by Haldar et al. (36) and Boyalla et al. (37), which compared CA and thoracoscopic surgical ablation (SA) in patients with long-standing persistent atrial fibrillation (LSPAF). Although these studies were not included in our analysis due to differences in intervention types, they reported that CA achieved comparable or superior outcomes in rhythm control, symptom relief, and cost-effectiveness, further supporting the utility of CA in complex AF populations.

While RhC and RC yielded intermediate benefits across several outcomes, they consistently underperformed relative to CA on composite endpoints. RhC also ranked lower in safety assessments, and RC did not demonstrate a clear advantage in tolerability. These findings underscore the need to tailor treatment choices to individual risk profiles and therapeutic objectives.

Heart failure-related hospitalizations represent a major burden for both patients and healthcare systems. The significant reduction in hospitalization rates observed with CA suggests its potential not only for rhythm control but also for stabilizing disease progression. This finding is consistent with results from CASA-AF trials by Haldar et al. (36) and Boyalla et al. (37), which reported improvements in quality-adjusted life years and symptom relief following CA.

Although CA and RhC showed trends toward reduced AF recurrence, statistical significance was not always achieved. Nonetheless, rhythm control remains clinically valuable. Maintaining sinus rhythm can alleviate AF-related symptoms, reduce thromboembolic risk, and enhance cardiac efficiency—particularly beneficial in patients with concurrent HF.

Despite CA’s superiority in clinical outcomes, it did not rank highest in safety metrics, highlighting the importance of careful patient selection, especially for those with multiple comorbidities. The CASA-AF program, including the health economic evaluations by Boyalla et al. (37) and Haldar et al. (38), demonstrated that CA was associated with fewer complications, shorter hospital stays, and superior cost-effectiveness compared to surgical ablation. Although our analysis did not reveal statistically significant differences in adverse event rates, SUCRA rankings suggested a slight safety edge for Rh + RC, indicating potential utility in high-risk groups.

A major limitation of our study is the absence of subgroup analyses based on HF phenotype (HFrEF, HFmrEF, HFpEF). Given the distinct pathophysiological mechanisms and differential treatment responses of these subtypes, this omission may obscure relevant heterogeneity. Unfortunately, most included RCTs lacked sufficient stratified data, precluding meaningful subgroup evaluation. Additionally, patients with HFmrEF were underrepresented, and tripartite classification was rarely reported. Future trials should address this gap by incorporating phenotype-specific data to guide more precise, individualized treatment recommendations.

A key strength of our analysis lies in its broad comparative scope, integrating both pharmacologic and invasive treatment strategies. By leveraging a Bayesian network meta-analytic approach, we synthesized a wide range of evidence to inform clinical decision-making and personalized care planning.

Our study employed rigorous methodological safeguards, including risk of bias assessment using ROB 2.0, GRADE grading of evidence certainty, and publication bias assessment via funnel plots. Most critical outcomes—such as all-cause mortality, hospitalization, and LVEF—were rated as having moderate to high certainty, supporting the credibility and clinical relevance of our conclusions.

Nonetheless, several limitations warrant consideration. First, the small number of direct head-to-head RCTs reduces confidence in certain indirect comparisons. Second, our inability to perform HF phenotype-specific analyses limits the applicability of findings to diverse patient populations. Third, most included trials lacked placebo control arms, constraining interpretation of absolute efficacy. Finally, the Bayesian network meta-analysis was conducted using Stata due to its accessibility and functional adequacy; however, it does not support reporting of heterogeneity indices (τ^2^, I^2^) or convergence diagnostics (e.g., trace plots, Gelman–Rubin statistics). Although we assessed consistency and robustness via SUCRA, the absence of formal MCMC diagnostics remains a methodological limitation. Future studies should consider dedicated Bayesian platforms like WinBUGS or JAGS to enhance analytic rigor.

Although we intended to present subgroup forest plots for key outcomes such as cardiovascular death, all-cause mortality, stroke, and heart failure hospitalization, we found that these endpoints were not uniformly reported across the included studies. Most trials only provided data on all-cause mortality and HF-related hospitalization, while cardiovascular death and stroke were either absent, inconsistently defined, or reported without sufficient subgroup stratification. This limitation restricted our ability to generate comprehensive and statistically valid forest plots for these outcomes.

Notably, only a few high-quality RCTs—such as CABANA (19, 20). Di Biase et al. (24) and MacDonald et al. (26)—provided a more complete set of safety and efficacy endpoints. However, the limited number of such studies and the heterogeneity in their definitions made subgroup synthesis and network connectivity infeasible for all four outcomes. Future clinical trials should strive for standardized endpoint reporting, especially for safety events like stroke and cardiovascular mortality, to support more robust subgroup analyses in network meta-analyses.

We echo recent consensus from the COMET initiative calling for standardized outcome reporting in AF-HF trials. Future studies should prioritize: Stratification by HF phenotype (HFrEF/HFpEF); Clear differentiation of stroke subtypes; Separate reporting of cardiovascular vs. non-cardiovascular death.

Although safety events such as major bleeding and stroke are of critical importance, they were inconsistently reported across the included studies. Only a limited number of trials provided event-level safety data with numerical details. Moreover, there was heterogeneity in definitions and follow-up periods. Therefore, we summarized available safety data in Supplementary Table 5 for reference but did not include these outcomes in the quantitative network meta-analysis due to the risk of biased estimation. We encourage future trials to adopt standardized reporting of adverse events to facilitate more comprehensive comparative evaluations.

## 5 Conclusion

This network meta-analysis provides robust evidence supporting the clinical superiority of CA over other evaluated strategies across critical outcomes, including enhanced cardiac function, reduced mortality, hospitalization, and AF recurrence among HF patients with concomitant AF. RhC and RC remain viable therapeutic alternatives for selected outcomes, while Rh + RC strategies may confer specific benefits in certain patient subsets, particularly in quality of life improvement. Clinical decision-making should integrate patient-specific comorbidities, procedural risks, and long-term outcomes data to optimize individualized care. Finally, our findings highlight the necessity for future large-scale, rigorously designed RCTs incorporating detailed subgroup analyses to further refine and enhance personalized treatment strategies for HF patients complicated by AF.

## Data Availability

The original contributions presented in the study are included in the article/Supplementary material, further inquiries can be directed to the corresponding author/s.
